# Versatile Bifunctional PYTA Derivatives for ^225^Ac Radiolabeling: A Comparison to Gold Standards

**DOI:** 10.2967/jnumed.125.270234

**Published:** 2025-09

**Authors:** Maxime Cheveau, Mathieu Moreau, Anna Grohmann, Sarah Robert, Alexandre Cochet, Bertrand Collin, Fréderic Boschetti, Sophie Poty, Franck Denat

**Affiliations:** 1 Université Bourgogne Europe, CNRS, Institut de Chimie Moléculaire de l’Université de Bourgogne, UMR 6302, Dijon, France;; 2 CheMatech, Dijon, France; and; 3 Centre Régional De Lutte Contre Le Cancer Georges François Leclerc, Dijon, France

**Keywords:** ^225^Ac, bifunctional chelators, radiochemistry, α-emitter, targeted α-therapy

## Abstract

We report the synthesis and evaluation of the first 3,6,10,13-tetraaza-1,8(2,6)-dipyridinacyclotetradecaphane-3,6,10,13-tetraacetic acid (PYTA) bifunctional chelators (BFCs) for ^225^Ac coordination. **Methods:** Three PYTA BFCs (PYTA-triacetate, PYTA-glutaric acid, and PYTA-pyridyl-ether) were synthesized. A comparative radiolabeling study with MACROPA, DOTA, and crown derivatives was performed. Conjugation to prostate-specific membrane antigen ligands and antibodies exemplified the applicability of these BFCs. Biodistribution and long-term stability of radiocomplexes were investigated in vivo. **Results:** PYTA derivatives demonstrated excellent radiochemical properties with quantitative radiolabeling under mild conditions (37 °C; low concentration) and exhibited prolonged in vitro stability. In vivo evaluation of radioimmunoconjugates confirmed the prolonged stability of PYTA conjugates, yielding results comparable to those seen with MACROPA, and revealed the instability of crown derivatives. **Conclusion:** PYTA emerges as a promising chelator for ^225^Ac, comparable to MACROPA, with the advantages of modular BFC synthesis.

## Visual Abstract

**Figure:**
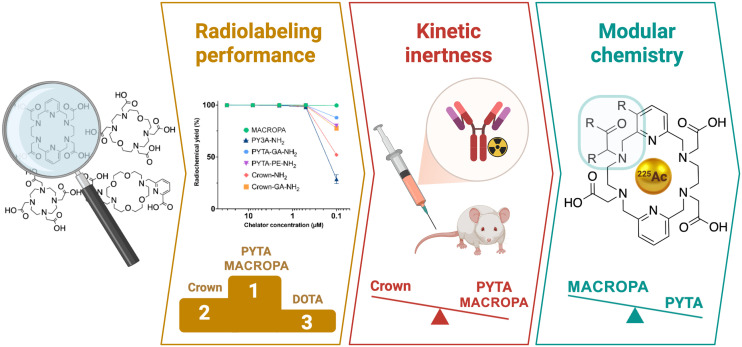

Targeted α-therapy (TAT) offers the potential for specific and effective cancer treatments (1,2). Among α-particle emitters, ^225^Ac has garnered attention for remarkable clinical outcomes when conjugated to prostate-specific membrane antigen (PSMA)–targeting ligands (3). However, its coordination chemistry remains poorly understood (4). Therefore, identifying a versatile chelator with a modular chemical structure for the insertion of a grafting moiety as well as the efficient and stable coordination of the radionuclide would leverage a major hurdle for ^225^Ac-TAT development. Although DOTA is widely used for ^225^Ac coordination, it demonstrates suboptimal radiolabeling efficiency, particularly when conjugated to antibodies (5–7). MACROPA has shown promise for ^225^Ac radiolabeling; however, its macrocyclic core is sensitive to modifications with reported compromised radiocomplex stability, therefore limiting functionalization opportunities to picolinate arms (8,9). Crown ether–based chelators have been explored with peptides but require further validation for long-term stability (10,11). More recently, 3,6,10,13-tetraaza-1,8(2,6)-dipyridinacyclotetradecaphane-3,6,10,13-tetraacetic acid (PYTA) emerged as a potential chelator for ^225^Ac, though no bifunctional derivatives have been reported (12,13). In this context, we synthesized and evaluated 3 novel PYTA bifunctional chelators (BFCs) with comparisons to MACROPA and crown-derived equivalent BFCs.

## MATERIALS AND METHODS

Information on chemistry, bioconjugation, radiochemistry, and in vitro and in vivo stability studies is provided in the supplemental materials, available at http://jnm.snmjournals.org (14–18).

Animal experiments were performed in compliance with the European Directive 2010/63/EU and Centre Régional De Lutte Contre Le Cancer Georges François Leclerc agreement (B 21 231 016 EA) after ethics approval. Athymic nude mice (Crl:Nu(NCr)-Foxn1nu, female, 6 wk; Charles River) received xenografts consisting of 6 × 10^6^ BxPC3 cells in 50:50 RPMI:matrigel on the right flank 3.5 wk before the biodistribution study to reach approximately 100–150 mm^3^ at the start of the study. Mice were randomized (3–4 per cohort, 5 cohorts per study, 68 total mice). Results are presented as mean ± SD. Statistical analyses were conducted using Dunnett T3 multiple comparisons test with adjusted *P* values.

## RESULTS

### Modular Chemical Approach

**FIGURE 1.**
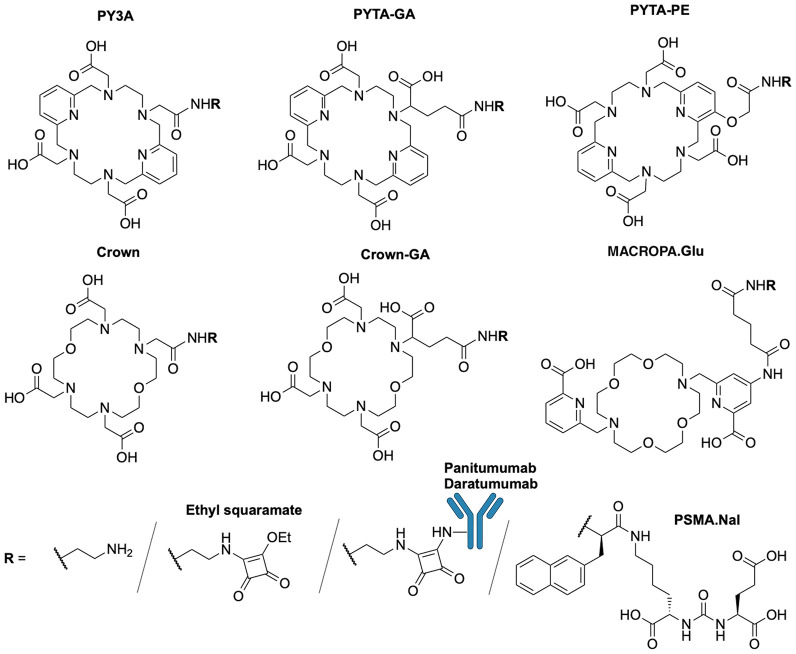
Representation of BFCs and bioconjugates.

Bifunctionalization of PYTA was performed using 3 strategies: conversion of a coordinating acetate, C-functionalization of an acetate arm, and functionalization of 1 pyridine, resulting in 3 PYTA-derived BFCs: PYTA-triacetate (PY3A), PYTA-glutaric acid (GA), and PYTA-pyridyl-ether (PE) (Fig. 1; Supplemental Schemes 1–3).

Although simple, acetate conversion into an amide induces a charge modification of PY3A derivatives, which can impact radiolabeling and radiocomplex stability. PYTA-GA synthesis required the mono-*N*-alkylation of the macrocyclic scaffold with a glutaric group, as previously described for DOTAGA synthesis (19). Finally, functionalization of 1 pyridine required a stepwise approach to produce PYTA-PE derivatives. 2,6-Bisalkyl-3-hydroxypyridine derivatives, previously used to functionalize pyridine-containing macrocycles (20,21), were applied here to enable easy and polyvalent functionalization via *O*-alkylation, while maintaining a N-hardness similar to the pyridine equivalent (22). The last 2 functionalization approaches were expected to preserve the radiocomplex geometry and overall charge. Moreover, our approaches prevented modification of macrocyclic carbon atoms that would generate a chiral center and yield diastereoisomers with different properties after radiolabeling (23). The same strategy was implemented for crown derivatives synthesis (Fig. 1; Supplemental Schemes 4 and 5).

### Radiolabeling of PYTA BFCs and Comparison with Gold Standards

**FIGURE 2.**
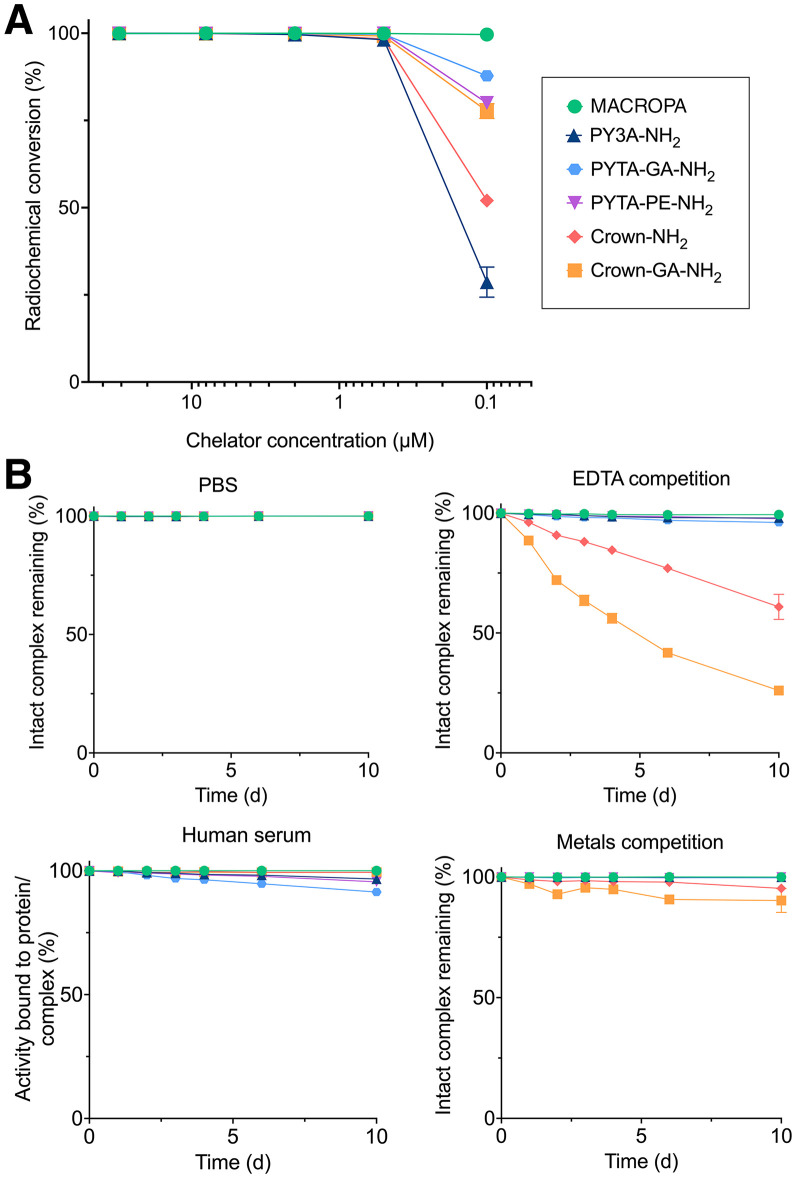
(A) Concentration-dependent radiolabeling of model chelators with ^225^Ac in NH_4_OAc 1 M buffer (pH, 6.0) at targeted molar activity of 116–119 MBq/µmol. (B) Stability of radiocomplexes in phosphate-buffered saline (PBS), human serum, PBS with 1,000 equivalents of ethylenediaminetetraacetic acid (EDTA), and PBS with 1 mM of Fe^3+^, Zn^2+^, Ca^2+^, Mg^2+^, and Cu^2+^.

PYTA BFCs and crown BFCs were coupled to ethylenediamine to assess their radiolabeling with ^225^Ac, mimicking the coordination when conjugated to a biovector. Radiolabeling of these model BFCs and MACROPA was evaluated in a concentration challenge assay with a concentration range (0.1–31 μM) that ensured coherence for future applications with small ligand or antibody conjugates and targeted molar activities (70–300 MBq/µmol) suitable for in vitro and in vivo applications (Fig. 2A; Supplemental Fig. 1). All tested BFCs exhibited excellent radiochemical conversion (>99%) down to 0.5 μM. At 0.1 μM, PY3A-NH_2_ and crown-NH_2_ exhibited the lowest radiochemical conversion, indicating that converting 1 coordinating acetate into acetamide reduces ^225^Ac coordination efficiency. All ^225^Ac radiocomplexes were stable (>90%) in phosphate-buffered saline and serum up to 10 d after radiolabeling (Fig. 2B). However, for crown-NH_2_ and crown-GA-NH_2_, instability was observed in the presence of ethylenediaminetetraacetic acid or metals competitors, highlighting transchelation and transmetalation.

**FIGURE 3.**
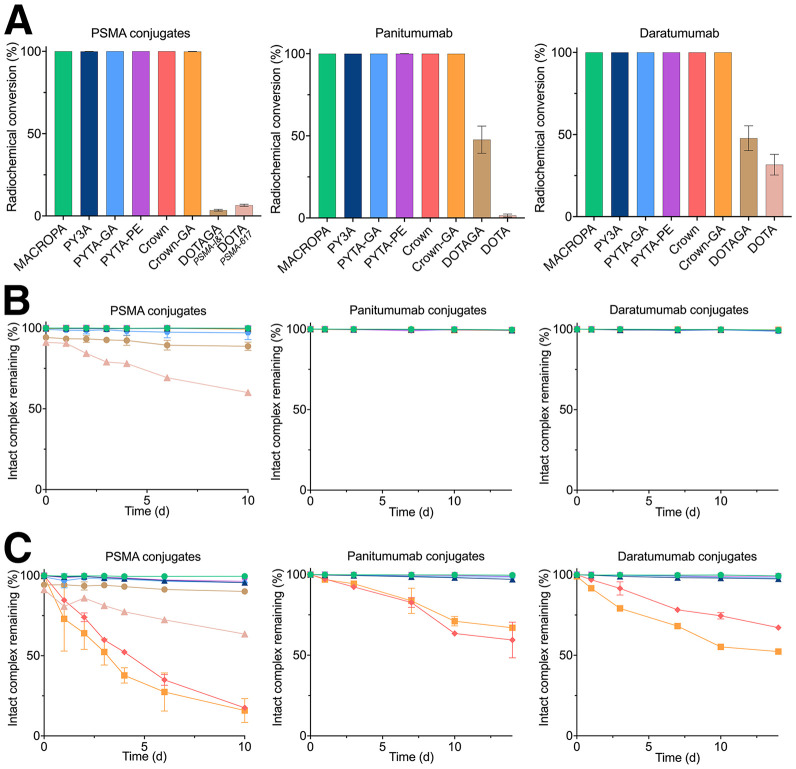
(A) Radiochemical conversion of PSMA, panitumumab, and daratumumab conjugates with ^225^Ac in NH_4_OAc 1 M buffer (pH 6.0) at 37 °C and targeted molar activities of 130 and 110 MBq/µmol for PSMA and antibody conjugates, respectively. Stability studies were performed in phosphate-buffered saline (B) and in competition with 1,000 equivalents of ethylenediaminetetraacetic acid (C).

To further demonstrate our BFCs versatility, PSMA inhibitor conjugates were synthesized and compared with clinical standards—PSMA-617 (DOTA conjugate) and PSMA I&T (DOTAGA conjugate). For MACROPA PSMA derivatives, functionalization of MACROPA-NH_2_-diester was performed through the addition of glutaric anhydride (MACROPA.Glu) to match with previous chelator bifunctionalization (Supplemental Scheme 6). Squaramate BFC derivatives were prepared for random lysine bioconjugation with 2 antibodies—panitumumab (anti–epidermal growth factor receptor [EGFR] IgG_2_) and daratumumab (anti-CD38 IgG_1_)—yielding conjugates with degrees of labeling from 2 to 7 (Supplemental Table 1). For a comprehensive comparison, DOTAGA- and DOTA-antibody conjugates were obtained. Radiolabeling performance of PSMA conjugates was excellent (>98%), except for PSMA-617 and PSMA I&T, which required heating at 90 °C (Fig. 3A; Supplemental Fig. 2). As with PSMA conjugates and previous reports, radiochemical conversions were below 50% for DOTA- and DOTAGA-antibody conjugates. Stability was not further investigated for these conjugates. Radiochemical conversions exceeded 98% for all other BFC conjugates. Overall, PYTA conjugates showed rapid incorporation of ^225^Ac and daughters within 5 min of incubation (Supplemental Table 2). A time-dependent decrease in intact complex was observed with PSMA-617 (Figs. 3B and 3C), suggesting instability of the DOTA conjugate. PSMA I&T demonstrated greater stability, suggesting a higher stability of DOTAGA conjugates and the deleterious effect of the conversion of 1 acetate to an amide. MACROPA conjugates and PYTA conjugates demonstrated excellent stability across all tested media. However, crown conjugates again exhibited transchelation when challenged with ethylenediaminetetraacetic acid. Based on this in vitro evaluation, the in vivo stability of 4 radiocomplexes (PYTA-GA, PYTA-PE, MACROPA, and crown-GA) was further investigated.

### In Vivo Stability of PYTA BFCs

**FIGURE 4.**
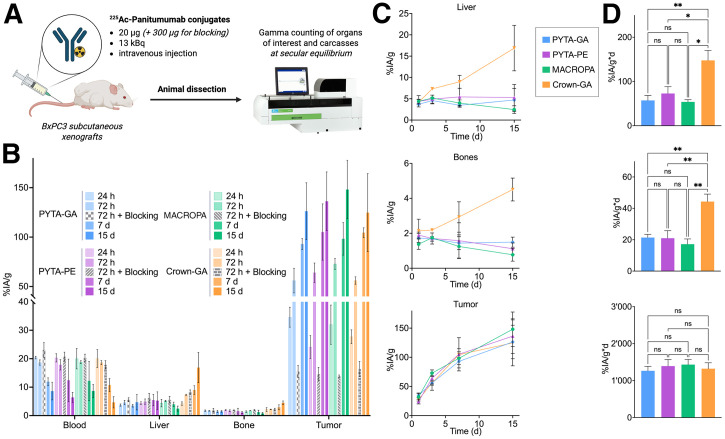
Biodistribution and in vivo stability evaluation of ^225^Ac-panitumumab conjugates in BxPC3 subcutaneous xenografts in athymic nude mice (*n* = 3 or 4 per cohort). (A) Overview of study. (B) Percentage of injected activity per gram of organ (%IA/g) of ^225^Ac conjugates in organs of interest. Evolution of %IA/g over time (C) and comparison of area under curve (%IA/g per day) (D) in liver, bone, and tumor. Statistical analyses were conducted using Dunnett T3 multiple comparisons test with adjusted *P* values. ns = nonsignificant; *P < 0.05; **P < 0.01.

To compare in vivo stability, we conducted a biodistribution study of ^225^Ac-panitumumab conjugates in mice bearing EGFR-positive (BxPC3) subcutaneous tumors over 15 d (Fig. 4A). Antibody conjugates were of interest because of their prolonged biologic circulation time. Uncoordinated ^225^Ac^3+^ distributes primarily to the liver, spleen, and bones (9). Therefore, attention was paid to these organs to assess the stability of our radiocomplexes. All 4 radioimmunoconjugates demonstrated strong EGFR-specific targeting, with increasing tumor uptake over 15 d and reduced uptake on blocking (Fig. 4B; Supplemental Figs. 4–7; Supplemental Table 3). Although PYTA-GA, PYTA-PE, and MACROPA-panitumumab conjugates exhibited similar pharmacokinetics, the crown-GA conjugate demonstrated increased uptake in the liver, spleen, and bone (Figs. 4C and 4D). The area under the curve or cumulated activity (percentage injected activity per gram × day) in the liver and bone was at least twice as high for the crown-GA conjugate compared with the 3 other conjugates (e.g., area under the curve for liver, 148 ± 22 for crown-GA vs. 73 ± 15 for PYTA-PE) (Fig. 4D), reflecting a slow release and redistribution of ^225^Ac^3+^. These observations underscore the long-term instability of ^225^Ac-crown radiocomplexes.

## DISCUSSION

Our results establish PYTA as an efficient chelator for long-lasting ^225^Ac coordination, offering a modular synthetic approach to BFCs through the introduction of conjugation moieties on either a pyridine or an acetate pendant arm. However, conjugation through 1 acetate group slightly hampers the radiolabeling efficiency of PY3A, and the stereocenter of PYTA-GA warrants further evaluation of its impact on stability and in vivo behavior. PYTA-PE appears to be the most promising BFC, and no chemical concern precludes it being produced on a larger scale. Radiolabeling and in vitro stability studies allowed a first comparison of PYTA BFCs to gold standards. Bioconjugation to a PSMA inhibitor and 2 antibodies (panitumumab and daratumumab) confirmed the efficient radiolabeling performance, with DOTA underperforming other chelators. In vivo evaluation of ^225^Ac-panitumumab conjugates highlighted the long-term instability of ^225^Ac-crown conjugates. This observation aligns with our in vitro results and trends reported in previous in vivo studies at earlier sacrifice times without comment from the authors (10). MACROPA and PYTA BFCs show prolonged stability in vivo. Rationalizing the kinetic inertness of our ^225^Ac radiocomplexes is challenging, as there are no reported crystal structures or density functional theory calculations available for crown complexes with actinium or lanthanide surrogates. However, differences in macrocyclic scaffold rigidity may explain the lower stability of crown versus PYTA complexes (12). In addition to its flexible scaffold, MACROPA forms highly inert complexes, likely due to its 2 picolinate κ^2^-coordinating pendant arms (24) and the distinct geometry of the actinium complex, with both pendant arms on the same face of the macrocycle in Δ(δλδ)(δλδ) and Δ(λδλ)(λδλ) conformers, stabilizing the radiometal within the macrocycle inner coordination sphere.

PYTA has been evaluated with several other radionuclides, including ^177^Lu, ^111^In, and ^44^Sc (12). Although the radiolabeling performance and stability with PYTA BFCs remains to be confirmed with those radionuclides, PYTA BFCs are expected to be highly versatile. This property should position PYTA as a promising platform for the development of next-generation theranostic agents.

## CONCLUSION

Our work highlights the potential of PYTA for the development of radiopharmaceuticals for ^225^Ac-TAT. Its structure enables easy derivatization to access various BFC versions, as exemplified herein. Our study demonstrated that PYTA BFCs outperform DOTA derivatives, which are suboptimal for ^225^Ac radiolabeling of antibodies. For the first time, to our knowledge, the performance of PYTA BFCs was demonstrated in vivo, with an exceptional stability of radiolabeled conjugates up to 15 d after injection, similar to that observed with MACROPA. Given their expected versatility with other radionuclides, which we are currently investigating, PYTA-based radiopharmaceuticals should become major players in the field of theranostics.

## DISCLOSURE

Support was provided by Association Nationale de la Recherche et de la Technologie, with a CIFRE fellowship granted to Maxime Cheveau, and by the French government through the program “Investissements d’Avenir” ANR-10-EQPX-05 − 01/IMAPPI Equipex. Sophie Poty receives Chaire Professeur Junior funding from the Agence Nationale de la Recherche. Maxime Cheveau, Anna Grohmann, and Sarah Robert are employees of CheMatech. Franck Denat is cofounder and shareholder of CheMatech. Fréderic Boschetti is CEO of CheMatech. No other potential conflict of interest relevant to this article was reported.

## Notes

KEYS POINTS **QUESTION:** Are PYTA BFCs valuable assets for ^225^Ac-TAT? **PERTINENT FINDINGS:** After evaluation of radiochemical performances and in vivo study, PYTA BFCs demonstrated outstanding properties and afforded results similar to those seen with MACROPA. **IMPLICATIONS FOR PATIENT CARE:** PYTA BFCs are expected to facilitate the development of ^225^Ac-TAT, enabling broader and safe access to treatment for patients.
